# Phytosynthesis via wasted onion peel extract of samarium oxide/silver core/shell nanoparticles for excellent inhibition of microbes

**DOI:** 10.1016/j.heliyon.2024.e24815

**Published:** 2024-01-20

**Authors:** Aisha A. Alshahrani, Laila S. Alqarni, Maha D. Alghamdi, Nasser F. Alotaibi, Shaima M.N. Moustafa, Amr M. Nassar

**Affiliations:** a Department of Chemistry, Faculty of Science, Al‐Baha University, P.O. Box 1988, Al‐Baha, 65799, Saudi Arabia; b Chemistry Department, College of Science, Imam Mohammad Ibn Saud Islamic University (IMSIU), Riyadh, Saudi Arabia; c Chemistry Department, College of Science, Jouf University, Sakaka, Saudi Arabia; d Biology Department, College of Science, Jouf University, Sakaka, Saudi Arabia

**Keywords:** Green, Onion peel, Sm_2_O_3_-NPs, Silver, Core/shell, Antimicrobial

## Abstract

The aqueous onion peel extract (OPE) was used to synthesize silver nanoparticles (Ag-onion), samarium oxide nanoparticles (Sm_2_O_3_-onion), and silver/samarium oxide core/shell nanoparticles (Ag@Sm_2_O_3_-onion). The produced nanoparticles were characterized by thermal gravimetric analysis (TGA), infrared spectra (FT-IR), absorption spectra (UV–Vis), energy band gap, X-ray photoelectron spectroscopy (XPS), X-ray diffraction (XRD), zeta potential, and transmission electron microscopy (TEM). OPE and NPs were tested for the disinfection of some water microbes. XRD analysis exhibited an amorphous structure of samarium oxide in both Sm_2_O_3_-onion and Ag@ Sm_2_O_3_-onion. The isolated bacteria from the water sample were *Bacillus subtilis* (OQ073500) and *Escherichia coli* (MW534699), while the isolated fungi were *Alternaria brassicae* (MZ266540), *Aspergillus flavus* (MT550030), *Aspergillus penicillioides* (MW957971), *Pythium ultimum* (MW830915), *Verticillium dahlia* (MW830379), *Fusarium acuminatum* (MZ266538), *Candida albicans* (MW534712), *and Candida parapsilosis* (MW960416). High levels of antimicrobial activity were seen in both the nanoparticles and the aqueous onion peel extract. Based on experimental results, Ag@Sm_2_O_3_ demonstrated the highest activity as an effective disinfectant, indicating the effectiveness of the modification process.

## 1 Introduction

Metal oxides have a significant role in many areas of physical, material, and biological science [1]. Metal oxides are created as a result of the propensity of metal ions to coordinate, which causes the development of a densely packed structure and the construction of a coordination sphere around the metal ions [2]. Scientists are especially interested in the many physical, magnetic, optical, and chemical properties of these compounds because metal oxides are highly sensitive to changes in composition and structure [3]. In contrast to the bulk metal oxide materials that are frequently available, nano metal oxides offer immense promise and can be applied in many areas of contemporary research [[4], [5], [6], [7]]. Metal oxide nanoparticles have significant uses in the broad category of nanomaterials that have been employed in biomedical applications, such as biosensing, immune therapeutics, drug delivery, regenerative medicine, bioimaging, wound healing, and so forth [[8], [9], [10]].

Sm_2_O_3_ is a rare earth oxide exhibit B-type centered monoclinic and C-type anion-deficient fluorite structures [11] and It has a band gap of about 4.3 eV [12] Samarium oxides, Sm_2_O_3_, have a variety of uses in environmental and catalytic science [[12], [13], [14], [15]]. It serves as a catalyst for the oxidative coupling of methane [16], the dehydration of alcohols [17], and the support for metal catalysts utilized in those reactions and serves as a refractory oxide in the creation of ceramic electrode cores [18].

Different physical and chemical methods, such as thermal decomposition, laser-induced deposition, sputtering, hydrogen-plasma assisted growth, sol-gel, hydrothermal, microwave, etc., have been used to produce Sm_2_O_3_ nanoparticles, nanorods, nanofibers, and nanowires [[19], [20], [21], [22]]. All of these techniques make use of risky chemicals or vacuum technology to reduce environmental problems. Therefore, a greener method of producing Sm_2_O_3_ is greatly valued. The primary component of the green synthesis method is the bio-oxidation/reduction of precursor metal salts utilizing plant extracts [23]. In addition, it has been established that Sm_2_O_3_ has excellent optoelectronic properties that make it suitable for biosensors, fuel cells, and antibacterial agents [24].

The current study deals with the biosynthesis of samarium oxide nanoparticles employing onion peel extract as a biroreductant. The characterization and antimicrobial activity of the nanoparticles were discussed.

## 2 Material and methods

### 2.1 Materials

The onion peels were obtained from market in Sakaka, Saudi Arabia. Sigma-Aldrich supplied the silver nitrate and samarium nitrate. Utilizing distilled water, the aqueous solutions were prepared.

### 2.2 Instruments

Thermo Scientific's Quattro S instrument was used for SEM photodetection. Fourier-transform infrared (FT-IR) spectral measurements have been performed using the spectrophotometer IRTracer-100 SHIMADZU. Using a copper radiation source, the XRD-7000 SHIMADZU was utilized to analyze X-ray diffraction (XRD) patterns. Thermal gravimetric analysis (TGA) was detected using the TGA-51SHIMADZU at a heating rate of 10 °C/min. A LABOMED-Spectro 99 UV–Vis double-beam 3200 was used to detect electronic spectra with wavelengths between 200 and 800 nm. applying an 80 kV JEOL GEM-1010 transmission electron microscope Using a JEOL GEM-1010 transmission electron microscope operating at 80 kV, the transmittance electron microscope (TEM) images were recorded. The Zeta potential measurements of the NPs were determined using the Brookhaven 90 plus Particle Size Analyzer (Brookhaven Instruments Corporation, American) at room temperature.

### 2.3 Synthesis of onion peels aqueous extract (OPE)

From the supermarket in Sakaka, Saudi Arabia, onion outer peels were gathered. To eliminate dirt and other contaminants from the onion peels, they were washed three times with distilled water, and then dried at room temperature. Then, 3g of onion peel were boiled in 500 mL for 30 min. The mixture was filtered, cooled, and stored until it was needed.

### 2.4 Green synthesis of nanoparticles

100 ml of OPE was added to an aqueous solution of 3 g of samarium nitrate in 50 mL of water. This mixture was then left to stirring for 2 h at 75 °C. The deep gray precipitate formed was filtered from the aqueous solution using filter paper designated as Whatman's No. 1, then calcined for 2h at 550 °C in a muffle furnace. The same procedures were used for Ag-onion and Ag@Sm_2_O_3_-onion with the use of silver nitrate 1.8 g/50 mL water and a 1:1 M ratio of a mixture in 100 mL water of silver nitrate (1.8g) and samarium nitrate (3g), respectively.

### 2.5 Antimicrobial experiments

A broth macro-dilution technique was used to determine the MIC and MBC or MFC. The determination of MBC (minimum bactericidal concentration) or MFC (minimum fungicidal concentration) was performed by preparing tubes that contain 2 ml (Nutrient broth or potato dextrose broth), 20 μl of each microbial culture, and six serial dilution concentrations (0.08, 0.16, 0.32, 0.64, 1.28, and 2.56 μg/mL) for OPE, Sm_2_O_3_-onion, Ag-onion, and Ag@Sm_2_O_3_-onion in each tube. After that, the tubes were incubated at 37 °C for 48 h [25]. The tests were performed in duplicate. The isolated bacteria were Bacillus subtilis (*B. subtilis* OQ073500), *Escherichia c*oli (*E. coli* MW534699) [26], and the isolated fungi were *Alternaria brassicae (A. brassicae MZ266540)* [27], *Aspergillus flavus (A. flavus MT550030)* [26], *Aspergillus penicillioides (A. penicillioides MW957971)* [28], *Pythium ultimum (P. ultimum MW830915)* [29], *Verticillium dahlia (V. dahlia MW830379)* [27], *Fusarium acuminatum (F. acuminatum MZ266538)* [27], *Candida albicans (C. albicans MW534712)* [26]*and Candida parapsilosis (C. parapsilosis MW960416)* were isolated from Agriculture and ground water, as well as sewage effluent in Sakaka. The antimicrobial activity was determined by the agar-well diffusion method [30]. The isolates were inoculated using nutrient broth (NB) for bacterial culture and Potato Dextrose Broth (PDB) medium for fungal culture, respectively. Each isolate was inoculated onto separate agar plates, and the wells (0.2-mm-diameter) were formed by using a cork borer. The wells were filled with 100 μl of tested compounds (OPE, Sm_2_O_3_-onion, Ag-onion, and Ag@Sm_2_O_3_-onion). The petri dishes were then incubated at 30 °C. The antimicrobial activity of the samples was measured by determining the diameter of the inhibition zone. All the experiments were repeated twice.

## 3 Results and discussion

### 3.1 Characterization of nanoparticles

#### 3.1.1 XRD study

Sm_2_O_3_ NPs were subjected to XRD analysis in order to determine their diffraction patterns. The findings revealed a diffusion band at lower scattering angles, pointing to the presence of low crystallinity structural and are typical of the amorphous structure and fine dimensions of biosynthesized Sm_2_O_3_. The amorphous nature of Sm_2_O_3_-onion is demonstrated by the broad hump appearing between 25° and 35° angles [31]. The results of the Ag-NPs XRD analysis showed prominent diffraction peaks found at 2θ of 77.6°, 64.6°, 44.6°, and 38.4° were well-aligned with the (311), (220), (200), and (111) planes of cubic metallic Ag (JCPDS #: 04–0783) [32]. The XRD of Ag@Sm_2_O_3_ nanocomposites shows that a similar pattern was seen for the Ag-NPs and Sm_2_O_3_, reflecting the presence of Ag on the Sm_2_O_3_ surface.

**Fig. 1:**
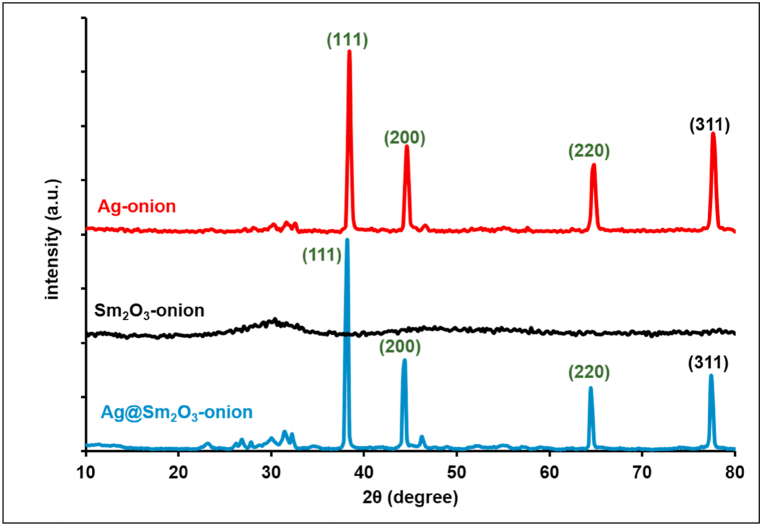
XRD of biosynthesized nanoparticles.

The Scherrer equation, eq. (1), was used to calculate the size of nanoparticles crystals [32].

(1) $$D=\frac{0.9.\lambda }{\beta .\cos\;\theta }$$

where θ is XRD angle, *β* is the half-maximum width and λ = 1.5418 Å is the wavelength of X-ray (for Cu Kα1). The crystals size was calculated to be 1.15 nm and 17.17 nm, and 18.11 nm for Sm_2_O_3_ and Ag, and Ag@Sm_2_O_3_, respectively, Fig. 1.

#### 3.1.2 TGA

**Fig. 2:**
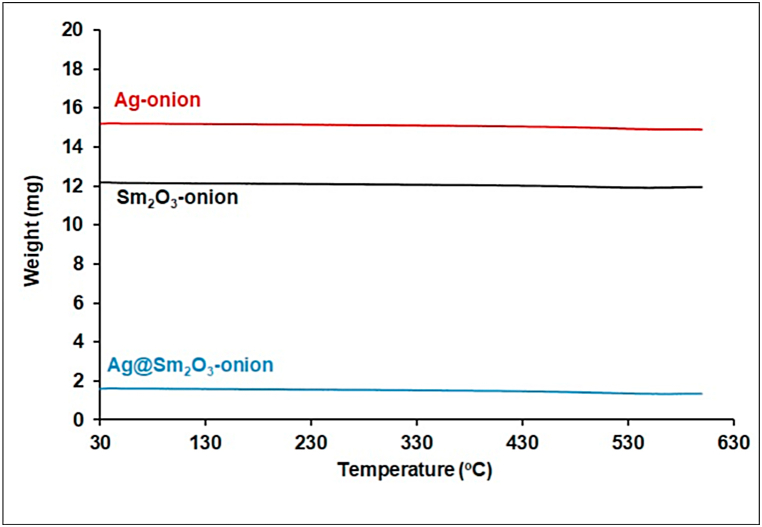
TGA of biosynthesized nanoparticles.

To ensure purity and thermal stability, the TGA of the synthesized nanoparticles was measured in the range of 30–600 °C, Fig. 2. The TGA curves show the absence of deprivation in the studied temperature range. This clears the pure weight of the biosynthesized NPs and confirms the removal of the organic biomolecules from onion peel extract during calcination.

#### 3.1.3 XPS analysis

**Fig. 3:**
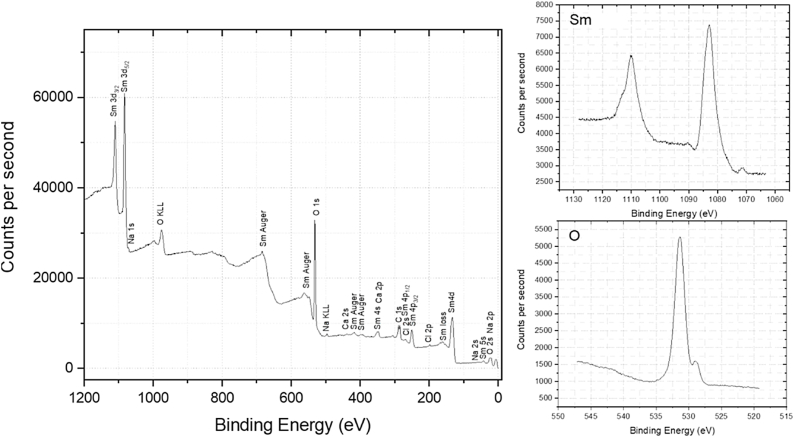
The XPS survey of Sm_2_O_3_-onion.

**Fig. 4:**
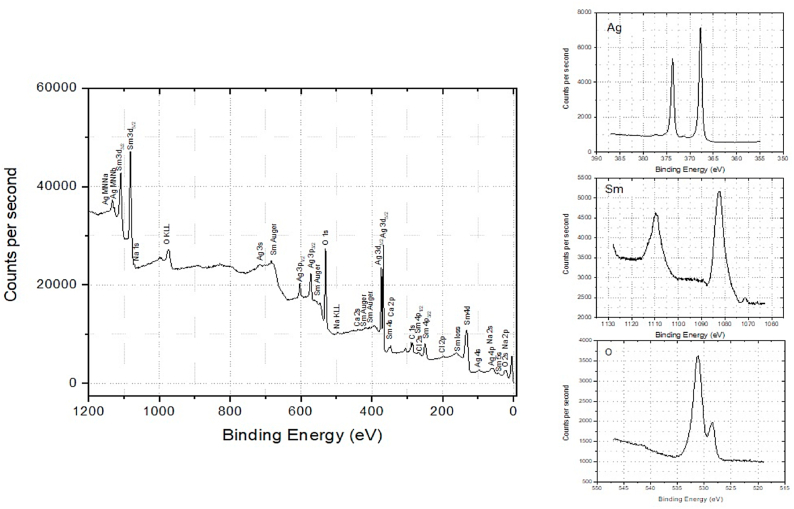
The XPS survey of Ag@Sm_2_O_3_-onion

The XPS was employed to evaluate chemical composition of the synthesized compound. In case of Sm_2_O_3_, XPS survey spectra in a wide energy range is applied and shown in Fig. 3.Two peaks present at 1081 eV and 1110 eV which can be attributed to 3d_5/2_ and 3d_3/2_ of Sm^3+^, respectively. It is indicated the presence of Sm^+3^ oxidation state in Sm_2_O_3_-onion [33,34]. Fig. 4 shows the XPS survey spectra of Ag@Sm_2_O_3_-onion. It reveals four peeks at bending energies at 373.90 eV, 367.90 eV, 1081 eV and 1110 eV which are attributed to Ag 3d_5/2_,Ag 3d _3/2_, Sm^3+^ 3d_5/2_ and Sm^3+^ 3d_3/2_, respectively [35]. The Sm_2_O_3_ and Ag@Sm_2_O_3_ spectra both showed the O_1s_ with binding energies of ∼528 eV and 531 eV.

#### 3.1.4 FT-IR

**Fig. 5:**
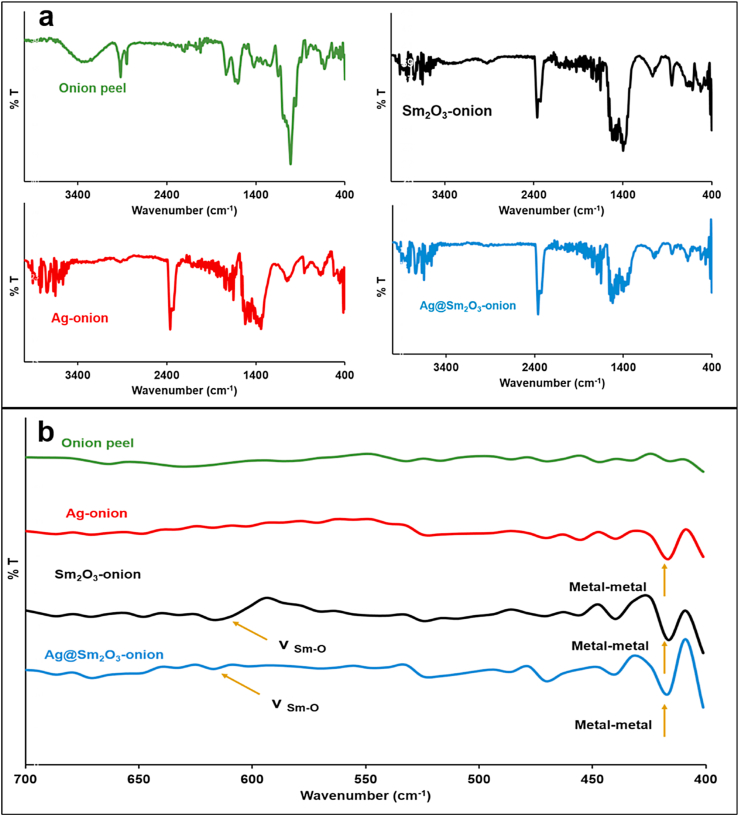
FTIR spectra of onion peel extract and biosynthesized nanoparticles: a) Whole spectra and b) expansion of range (400–700 cm^−1^).

FT-IR spectra of solid onion peels and biosynthesized nanoparticles were measured in the 400-4000 cm^−1^ spectral region, Fig. 5a. The FTIR spectrum of onion peel reveals distinct peaks due to the presence of bioorganic molecules. The broad band at 3287 cm^−1^ is assignable to OH stretching vibration indicates the presence of free OH from flavonoids and phenols [36]. The width of this band is due to intermolecular hydrogen bonds between the organic molecules. The weak bands at 2850 and 2950 cm^−1^ are assignable to the stretching of the aliphatic and aromatic C–H bond, respectively. The N–H (amine) bends and the ring C–C stretch of phenyl corresponds to bands at 1680 cm^−1^ and 1600 cm^−1^, respectively [37]. The functional groups from carbohydrates are what cause the wide peak at around 1000 cm^−1^. Also, the band at 1800 cm^−1^ is assignable to –C=O group [37] These bands have all disappeared in the NPs IR spectra, indicating the removal of all organic molecules and the purity of NPs. The spectra of NPs show metal-metal interaction is responsible for the appearance of new bands in the 400–500 cm^−1^ region [5]. The spectra of Sm_2_O_3_-onion and Ag@Sm_2_O_3_-onion show band around 590-620 cm^−1^, which can be assigned to the Sm–O vibration [38], Fig. 5b.

#### 3.1.5 Ultraviolet–visible (UV–vis) spectroscopy

**Fig. 6:**
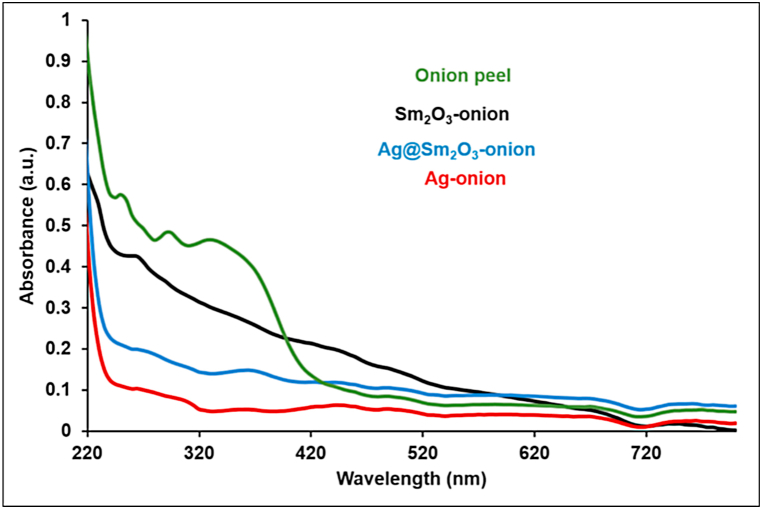
UV–vis spectra of onion peel extract and of biosynthesized nanoparticles.

The UV–Vis spectroscopic analysis is a useful technique for demonstrating the presence of metal nanostructures [[39], [40], [41]]. The optical absorption characteristics of onion peel water extract and nanoparticles were measured using the UV–vis technique by recording the spectra in the wavelength range of 200–600 nm, as shown in Fig. 6. The UV–Vis spectrum of red onion extract revealed peaks at 255 nm, 285 nm, and 350 nm, signifying the presence of organic molecules such as flavanols, quercetin and anthocyanins [42,43]. The UV–vis spectra of Ag NPs, Sm_2_O_3_ NPs, and Ag@Sm_2_O_3_ nanocomposites showed distinct absorption peaks at 275 and 375 nm due to the biosynthesized nanoparticles distinctive surface plasmon resonance (SPR) [39,44]. The peak around 450 nm is characteristic and indicates the reduction of Ag ^+^ ions to Ag^0^ [45].

#### 3.1.6 Energy band gap

**Fig. 7:**
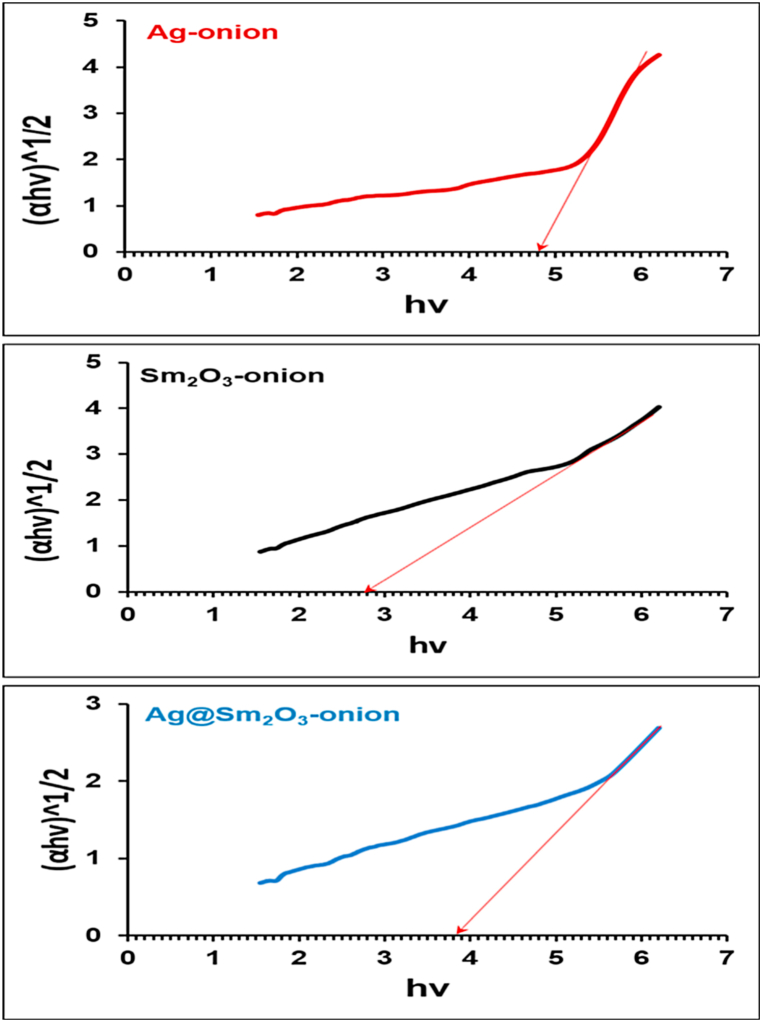
Tuac's plot for biosynthesized nanoparticles.

Fig. 7 shows the optical band gap for the indirect transition is found using Tuac's equation, where A is the independent energy constant, Eg is the determined band gap, and α is the absorption coefficient [46]. For Ag-onion, Sm_2_O_3_-onion, and Ag@Sm_2_O_3_-onion, the obtained band gaps are 4.8 eV, 2.8.eV, and 3.7 eV, respectively. The increasing band gap of Ag-onion may be due to the quantum confinement effect. The band gap widens, and the number of orbital energy levels decreases with the smaller crystal size [47]. Also, the value of the band gap of Sm_2_O_3_ is in the range of ⁓ 3–4 eV, indicating the presence of an amorphous structure [48].

#### 3.1.7 Zeta potential measurements

**Fig. 8:**
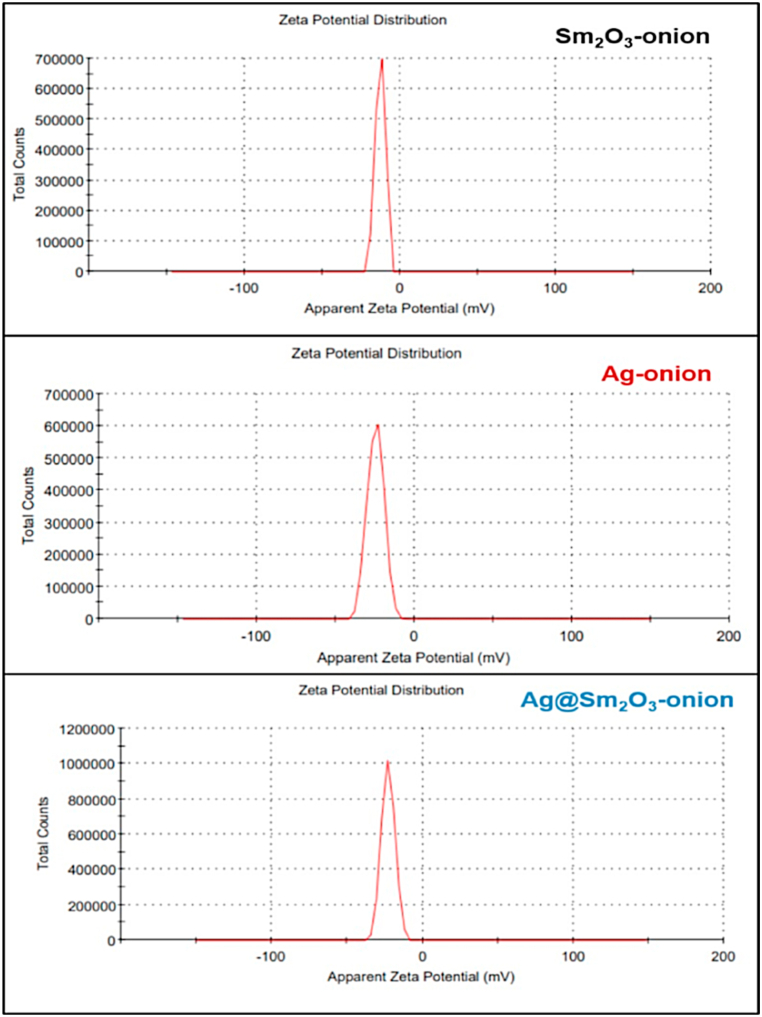
Zeta potential distribution of biosynthesized nanoparticles.

Zeta potential is an important characteristic to take into consideration when evaluating system stability, effective surface electric charge, and dispersion of nanoparticles [49]. Fig. 8 shows the zeta potential results of Ag-onion, Sm_2_O_3_-onion, and Ag@Sm_2_O_3_-onion, which are −24.6 mV, −12.7 mV, and −22.8 mV, respectively. This verifies that the surface of the biosynthesized NPs has negative-charged groups. The negatively charged surface helps to regulate the size of the formed NPs and prevents them from aggregating [50].

#### 3.1.8 TEM studies

**Fig. 9:**
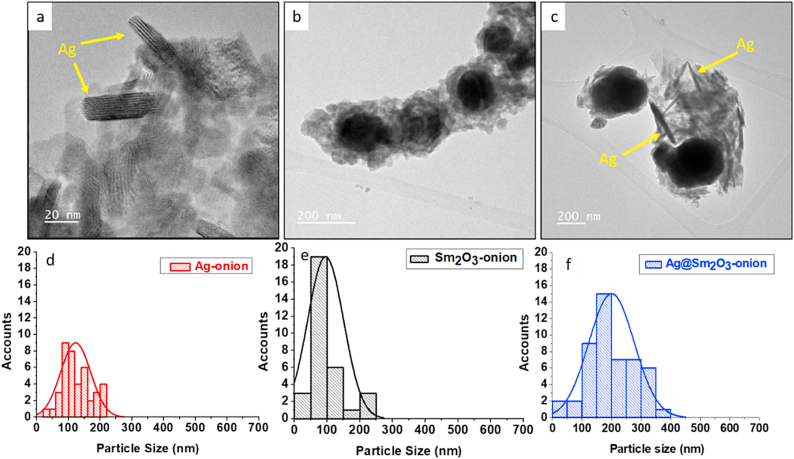
TEM images and particle size distribution of synthesized nanoparticles, a,d) Ag-onion, b,e) Sm_2_O_3_-onion and c,f) Ag@ Sm_2_O_3_-onion.

To present the morphology of the particles in more detail, TEM analysis is carried out for the synthesized NPs as shown in Fig. 9. It can be shown that there are some needles in Fig. 9a. It is attributed to forming Ag nanoparticles with needles shape. Fig. 9b shows spherical shape of Sm_2_O_3_. The morphology of the samarium oxide/silver core/shell structure can be illustrated in Fig. 9c. It appears as a spherical core (Sm_2_O_3_) surrounded by a needle shell (Ag), confirming the presence of a Sm_2_O_3_/Ag core/shell structure.

#### 3.1.9 Mechanism for the metal ions reduction to metallic nanoparticles

**Fig. 10:**
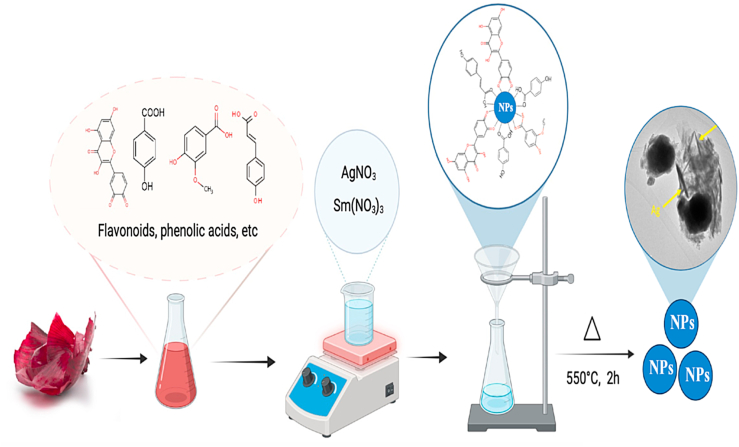
Suggested mechanism for the formation of nanoparticles via onion peel extract.

It is well known that plant extracts are abundant in phytochemical components such as sugars, flavonoids, glycosides, phenolic acids, gallic acids, and saponins [51,52]. Because of their potential to produce reactive oxygen species (ROS), these constituents are known as bioactive molecules [53]. Onion peel aqueous extract contains flavonoids such as quercetin, along with protocatechuic acid, *p*-hydroxybenzoic acid, vanillinic acid, *p*-cumaric acid, myricetin, kaempferol, and isorhamnetin-3-glucoside [54,55]. Fig. 10 displays the schematic reaction of metal salts with organic molecules in aqueous onion peel extract, like flavonoids and phenolic acids. The reaction of cations with phytochemicals results in complex formation that stabilizes the nanoparticle. The complex is formed due to a chemical connection between cations and the electron-rich organic molecules in plant extract. This mechanism is proposed for biosynthesized nanoparticles via other plant extracts [56] The complex formation is responsible for the synthesis, growth, and stabilization of nanoparticles [57].

### 3.2 Biological studies

#### 3.2.1 Antimicrobial activity

**Fig. 11:**
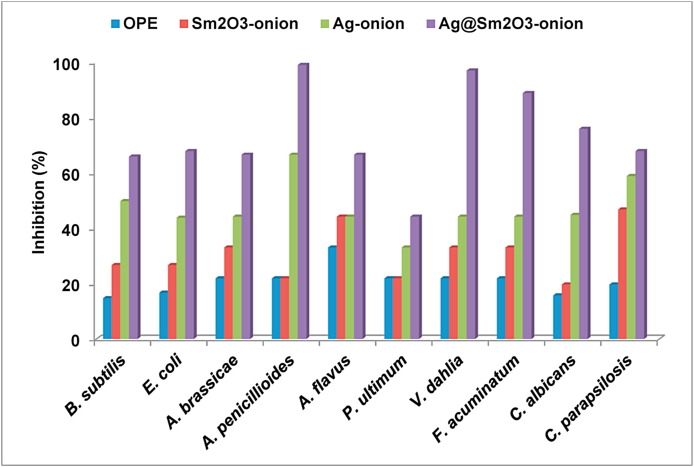
Antimicrobial activity of onion peel extract and of biosynthesized nanoparticles.

**Fig. 12:**
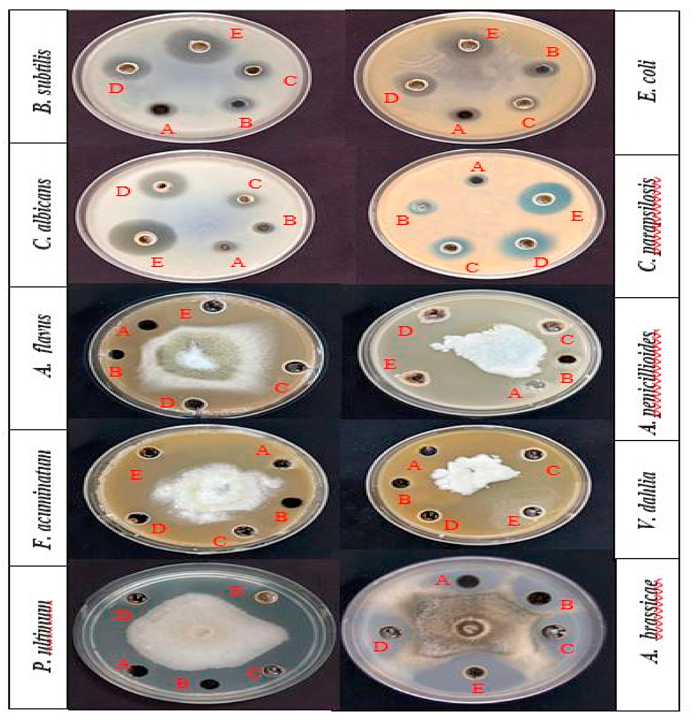
Effect of OPE (A), (B) Control (ampicillin or miconazole), Sm_2_O_3_-onion (C), Ag-onion (D), and Ag@Sm_2_O_3_-onion (E) on the growth of tested microbes, under dark condition at 30 °C.

By employing the broth macro-dilution approach in conjunction with the normal serial dilution method, MIC, MBC, and MFC values of various concentrations of 0.08, 0.16, 0.32, 0.64, 1.28, and 2.56 μg/mL were reported against tested microbes [19]. Table 1 provides a summary of the findings. For OPE, Sm_2_O_3_-onion, Ag-onion, and Ag@ Sm_2_O_3_-onion. The antimicrobial results are shown in Fig. 11, Fig. 12 as illustrations. According to the results, Ag@Sm_2_O_3_-onion showed the best activity among the studied nanoparticles.

**Table 1:** Antimicrobial activity of OPE, Sm_2_O_3_-onion, Ag-onion, and Ag@Sm_2_O_3_-onion against tested microbes at 30 °C in dark.

Microbial strain	OPE			Sm_2_O_3_-onion			Ag-onion			Ag@Sm_2_O_3_-onion		
	MIC	MBC	MFC	MIC	MBC	MFC	MIC	MBC	MFC	MIC	MBC	MFC
*B. subtilis*	1.28	2.65	–	2.65	2.65	–	0.64	0.64	–	0.16	0.32	–
**E. coli**	1.28	2.65	–	1.28	2.65	–	0.32	0.64	–	0.16	0.32	–
*A. brassicae*	1.28	–	2.65	2.65	–	2.65	0.32	–	0.32	0.16	–	0.32
*A. penicillioides*	2.65	–	2.65	1.28	–	2.65	0.32	–	0.32	0.32	–	0.32
*A. flavus*	2.65	–	2.65	2.65	–	2.65	0.64	–	0.64	0.32	–	0.64
*P. ultimum*	1.28	–	2.65	1.28	–	2.65	0.32	–	0.32	0.16	–	0.16
*V. dahlia*	2.65	–	2.65	1.28	–	1.28	0.32	–	0.64	0.16	–	0.32
*F. acuminatum*	2.65	–	2.65	2.65	–	2.65	0.64	–	0.64	0.32	–	0.32
*C. albicans*	1.28	–	2.65	1.28	–	2.65	0.32	–	0.64	0.16	–	0.32
*C. parapsilosis*	2.65	–	2.65	2.65	–	2.65	0.64	–	0.32	0.32	–	0.16

MIC: minimum inhibitory concentration; MFC: minimum fungicide concentration, and MBC: minimum bactericidal concentration. A Result expressed as μg/mL.

#### 3.2.2 Mechanism of antimicrobial activity

**Fig. 13:**
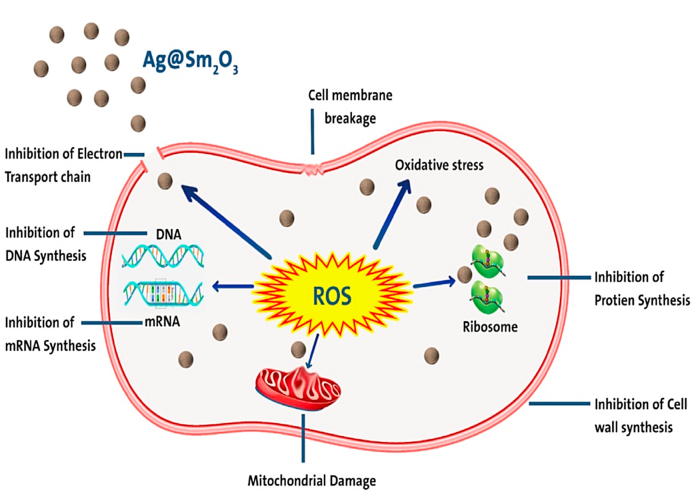
Suggested mechanism of antimicrobial activity.

Various metal and metal oxide NPs have reportedly been created and evaluated by numerous research organisations. Fig. 13 demonstrated the various ways that NPs engage with microbes, and each type's mode of operation is distinct [45]. Different metal and metal oxide NPs interact with nucleic acids to form complexes with them, and subsequently, these complexes are shown with nucleosides to show antimicrobial action. Nanoparticles changed the permeability of the membrane, as seen by the passage of carbohydrates, proteins, and nuclear material through the damaged membrane. Due to the NPs' improved adherence to the cytoplasm and cell membrane as well as the breakdown of the microbial casings, the permeability of the microbial cell membrane increased. Adenosine triphosphate (ATP) release may be inhibited when reactive oxygen species (ROS) are produced more quickly as a result of the generation of free ions. The intracellular and extracellular components of the membrane may separate as a result of denaturation of the microbial cell membrane, which also results in cell lysis [58,59]. In this work, Ag@Sm_2_O_3_-onion has potent antimicrobial activity that may be attributed to the nanoparticles' attachment to microbial surfaces, cell membrane rupture, modification of the respiration chain, and other permeability-dependent activities, and ultimately destruction of microbial significant proteins.

Table 2 exhibits the comparison of the bioactivity of biosynthesized nanoparticles with other similar compounds.

**Table 2:** Comparison of the antimicrobial activity of Ag@Sm_2_O_3_ and the previously reported similar nanoparticles.

NPs	Bioactivity	Effect	Ref
Sm/Ag/TiO_2_	Antibacterial	Sm/Ag/TiO_2_ shows high antibacterial characteristics, for the white beads coccus, the lowest bacteriostatic concentration of Sm/Ag/TiO_2_ was 200 μg/mL, while the minimum bactericidal concentration was 2 × 10^4^ μg/mL	[60]
Sm_2_O_3_-NPs	Antibacterial	1-Butyl 3-methyl imidazolium hexafluorophosphate ionic liquid-assisted Sm_2_O_3_ NPs were examined in various concentration ratios of 50, 100, and 150 mL for their antibacterial efficacy against *S. aureus* and *E. coli* and showed a zone of inhibition of 15, 17, and 18 mm against *S. aureus* and 17, 20, and 22 mm against *E. coli*, respectively.	[61]
Sm_2_O_3_ NPs	Antibacterial	*S. aureus* had the lowest MIC value (3.12 μg/mL), and *P. P. aeruginosa* had the highest MIC value (25 μg/mL).	[62]
Ag@Sm_2_O_3_	Antimicrobial	Ag@Sm_2_O_3_-onion showed strong inhibition at concentrations of 0.16 μg/mL against gram-positive and gram-negative tested bacteria and 0.16⁓ 0.32 μg/mL for tested fungi.	Current work

## 4 Conclusion

In order to fabricate silver nanoparticles, samarium oxide nanoparticles, and silver/samarium oxide core/shell nanoparticles, an aqueous extract of onion peels was treated with silver nitrate, samarium nitrate, and a combination of silver nitrate and samarium nitrate, respectively. Several physicochemical methods were used to confirm the formation of nanoparticles. The antimicrobial capacity of aquatic bacterial and fungal strains that are waterborne pathogens was investigated. The onion peel aqueous extract and nanoparticles both showed high levels of antimicrobial activity. Ag@Sm_2_O_3_ was the most effective antimicrobial agent, showing the greatest activity in disinfection against all isolated microorganisms.

## CRediT authorship contribution statement

**Aisha A. Alshahrani:** Writing – review & editing, Writing – original draft, Data curation. **Laila S. Alqarni:** Writing – review & editing, Writing – original draft, Data curation. **Maha D. Alghamdi:** Writing – review & editing, Writing – original draft, Data curation. **Nasser F. Alotaibi:** Writing – review & editing, Writing – original draft, Funding acquisition. **Shaima M.N. Moustafa:** Writing – review & editing, Writing – original draft, Data curation. **Amr M. Nassar:** Writing – review & editing, Supervision, Methodology.

## Declaration of competing interest

The authors declare that they have no known competing financial interests or personal relationships that could have appeared to influence the work reported in this paper.
